# Cinnarizinium 3,5-dinitro­salicylate

**DOI:** 10.1107/S1600536812011518

**Published:** 2012-03-24

**Authors:** Alaloor S. Dayananda, Hemmige S. Yathirajan, Thomas Gerber, Eric Hosten, Richard Betz

**Affiliations:** a University of Mysore, Department of Studies in Chemistry, Manasagangotri, Mysore 570 006, India; b Nelson Mandela Metropolitan University, Summerstrand Campus, Department of Chemistry, University Way, Summerstrand, PO Box 77000, Port Elizabeth 6031, South Africa

## Abstract

The title compound [systematic name: 4-diphenyl­methyl-1-(3-phenylprop-2-en-1-yl)-piperazin-1-ium 2-carb­oxy-4,6-dinitro­pheno­late], C_26_H_29_N_2_^+^·C_7_H_3_N_2_O_7_^−^, is the dinitro­salicylate salt of a tertiary amine. Deprotonation of the carb­oxy­lic acid group occurred on the phenolic hy­droxy group. The diaza­cyclo­hexane ring adopts a chair conformation. Intra­molecular O—H⋯O and inter­molecular C—H⋯O and N—H⋯O hydrogen bonds are observed. The N—H⋯O hydrogen bonds are bifurcated at the H atom and connect the cinnarizinium and 3,5-dinitro­salicylate ions together. Inter­molecular C—H⋯O hydrogen bonds connect the components into layers perpendicular to the crystallographic *a* axis.

## Related literature

For pharmaceutical background to cinnarizine, see: Barrett & Zolov (1960 ▶). For related structures, see: Bertolasi *et al.* (1980 ▶); Smith *et al.* (2001 ▶); Jasinski *et al.* (2011 ▶). For puckering analysis, see: Cremer & Pople (1975 ▶). For graph-set analysis of hydrogen bonds, see: Etter *et al.* (1990 ▶); Bernstein *et al.* (1995 ▶).
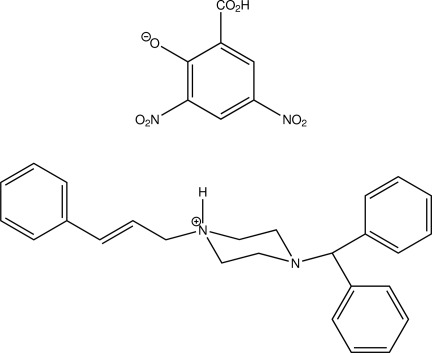


## Experimental

### 

#### Crystal data

C_26_H_29_N_2_^+^·C_7_H_3_N_2_O_7_^−^
*M* *_r_* = 596.63
Monoclinic, 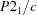

*a* = 14.5648 (3) Å
*b* = 12.9374 (3) Å
*c* = 16.1619 (3) Å
β = 103.900 (1)°
*V* = 2956.22 (11) Å^3^
*Z* = 4
Mo *K*α radiation
μ = 0.10 mm^−1^
*T* = 200 K
0.51 × 0.26 × 0.17 mm

#### Data collection

Bruker APEXII CCD diffractometer
Absorption correction: multi-scan (*SADABS*; Bruker, 2008 ▶) *T*_min_ = 0.932, *T*_max_ = 1.000
29552 measured reflections
7344 independent reflections
6023 reflections with *I* > 2σ(*I*)
*R*_int_ = 0.015

#### Refinement

*R*[*F* ^2^ > 2σ(*F* ^2^)] = 0.051
*wR*(*F* ^2^) = 0.148
*S* = 1.03
7344 reflections
401 parameters
H atoms treated by a mixture of independent and constrained refinement
Δρ_max_ = 0.85 e Å^−3^
Δρ_min_ = −0.35 e Å^−3^

### 

Data collection: *APEX2* (Bruker, 2010 ▶); cell refinement: *SAINT* (Bruker, 2010 ▶); data reduction: *SAINT*; program(s) used to solve structure: *SHELXS97* (Sheldrick, 2008 ▶); program(s) used to refine structure: *SHELXL97* (Sheldrick, 2008 ▶); molecular graphics: *ORTEP-3* (Farrugia, 1997 ▶) and *Mercury* (Macrae *et al.*, 2008 ▶); software used to prepare material for publication: *SHELXL97* and *PLATON* (Spek, 2009 ▶).

## Supplementary Material

Crystal structure: contains datablock(s) I, global. DOI: 10.1107/S1600536812011518/rn2101sup1.cif

Supplementary material file. DOI: 10.1107/S1600536812011518/rn2101Isup2.cdx

Structure factors: contains datablock(s) I. DOI: 10.1107/S1600536812011518/rn2101Isup3.hkl

Supplementary material file. DOI: 10.1107/S1600536812011518/rn2101Isup4.cml

Additional supplementary materials:  crystallographic information; 3D view; checkCIF report

## Figures and Tables

**Table 1:** Hydrogen-bond geometry (Å, °)

*D*—H⋯*A*	*D*—H	H⋯*A*	*D*⋯*A*	*D*—H⋯*A*
N1—H71⋯O1^i^	0.89 (2)	1.99 (2)	2.8105 (18)	153.5 (17)
N1—H71⋯O2^i^	0.89 (2)	2.36 (2)	3.037 (2)	132.4 (16)
O7—H7⋯O1	0.84	1.75	2.507 (2)	149
C3—H3*A*⋯O6^ii^	0.99	2.40	3.341 (2)	160
C32—H32⋯O5	0.95	2.52	3.453 (2)	166

Symmetry codes: (i) 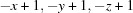; (ii) 
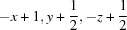
.

**Fig. 1.**
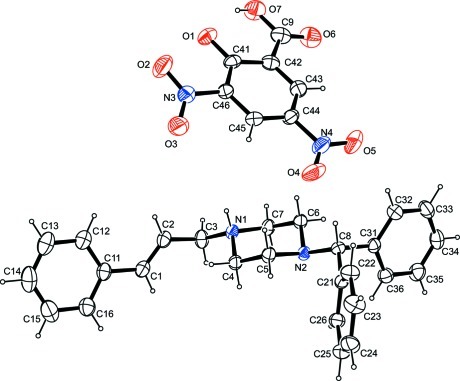
The molecular structure of the title compound, with atom labels and anisotropic displacement ellipsoids (drawn at 50% probability level).

**Fig. 2.**
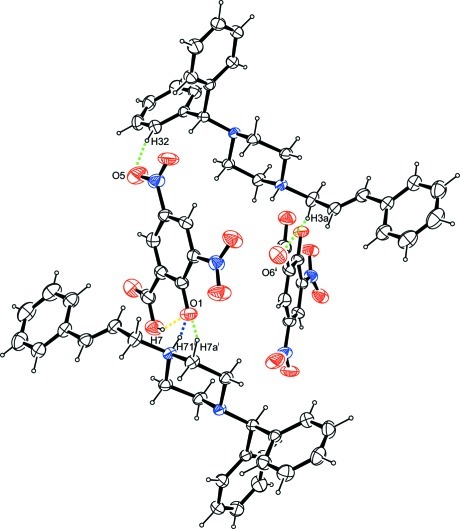
Intermolecular contacts, viewed approximately along [1 1 1]. For clarity, only a selection of intra- and intermolecular contacts is depicted. Yellow dashed lines denote intramolecular hydrogen bonds, blue dashed lines denote intermolecular hydrogen bonds and green dashed lines indicate C–H···O contacts. Symmetry operators: i -x + 1, -y + 1, -z + 1; ii -x + 1, y + 1/2, -z + 1/2.

## supplementary crystallographic information

### Comment

Cinnarizine is an antihistamine which is mainly used for the control of nausea and vomiting due to motion sickness. A clinical evaluation of cinnarizine in various allergic disorders is published (Barrett & Zolov, 1960). The crystal structures of some related compounds such as cyclizine hydrochloride (Bertolasi *et al.*, 1980), guanidinium 3,5-dinitrosalicylate (Smith *et al.*, 2001) and cinnarizine dipicrate (Jasinski *et al.*, 2011) have been reported.

Deprotonation occurred at the phenolic hydroxy group while the carboxyl group remains in its protonated state. Protonation occurred on the nitrogen atom bearing the vinylic substituent. The 1,4-diazacyclohexane moiety adopts a ^4^*C*_1_ (^N2^*C*_N1_) conformation (Cremer & Pople, 1975). The least-squares planes defined by the carbon atoms of the aromatic moieties on the diphenylmethyl substituent enclose an angle of 66.06 (8)° and intersect with the plane defined by the phenyl group bonded to the vinylic substituent at angles of 16.76 (9)° and 72.01 (9)°, respectively. The least-squares planes defined by the individual phenyl groups of the cation enclose angles of 37.54 (9)°, 53.70 (8)° and 79.26 (8)° with the corresponding plane of the carboxylic acid (Fig. 1).

In the crystal, intramolecular O—H···O and intermolecular C—H···O and N—H···O hydrogen bonds in are observed. The N–H···O hydrogen bonds are bifurcated at H7. A graphical representation in terms of donor and acceptor atoms for a selection of these contacts is given in Figure 2. In terms of graph-set analysis, (Etter *et al.*, 1990; Bernstein *et al.*, 1995), the descriptor for the hydrogen bonds is *S*(6)*DD* on the unary level while the C–H···O contacts necessitate a *DD* descriptor on the same level. The C–H···O hydrogen bonds connect the components together into layers perpendicular to the crystallographic *a* axis (Fig. 2). The shortest intercentroid distance between two aromatic systems was measured at 4.2848 (11) Å and is apparent between the phenyl group conjugated to the vinylic system and the aromatic ring of the dinitrosalicylate anion.

### Experimental

Cinnarizine (3.68 g, 0.01 mol) and 3,5-dinitrosalicylic acid (2.28 g, 0.01 mol) were dissolved in hot acetone and stirred over a heating magnetic stirrer for few minutes (330 K). The resulting solution was allowed to cool slowly at room temperature. The salt formed was filtered and dried in a vaccum desiccator over phosphorous pentoxide. The compound was recrystallized from a mixture of DMSO and acetonitrile (*v*:*v* = 1:1) by slow evaporation (m.p.: 383 K).

### Refinement

Carbon-bound H atoms were placed in calculated positions (C—H 0.95 Å for vinylic and aromatic C atoms, C—H 0.99 Å) and were included in the refinement in the riding model approximation, with *U*(H) set to 1.2*U*_eq_(C). The nitrogen-bound H atom was located on a difference Fourier map and their coordinates as well as isotropic displacement parameters were refined freely.

### Crystal data

**Table:**

C_26_H_29_N_2_^+^·C_7_H_3_N_2_O_7_^−^	*F*(000) = 1256
*M**_r_* = 596.63	*D*_x_ = 1.341 Mg m^−^^3^
Monoclinic, *P*2_1_/*c*	Melting point: 383 K
Hall symbol: -P 2ybc	Mo *K*α radiation, λ = 0.71073 Å
*a* = 14.5648 (3) Å	Cell parameters from 9909 reflections
*b* = 12.9374 (3) Å	θ = 2.3–28.3°
*c* = 16.1619 (3) Å	µ = 0.10 mm^−^^1^
β = 103.900 (1)°	*T* = 200 K
*V* = 2956.22 (11) Å^3^	Block, yellow
*Z* = 4	0.51 × 0.26 × 0.17 mm

### Data collection

**Table:**

Bruker APEXII CCD diffractometer	7344 independent reflections
Radiation source: fine-focus sealed tube	6023 reflections with *I* > 2σ(*I*)
Graphite monochromator	*R*_int_ = 0.015
φ and ω scans	θ_max_ = 28.3°, θ_min_ = 2.0°
Absorption correction: multi-scan (*SADABS*; Bruker, 2008)	*h* = −19→19
*T*_min_ = 0.932, *T*_max_ = 1.000	*k* = −17→17
29552 measured reflections	*l* = −21→15

### Refinement

**Table:**

Refinement on *F*^2^	Primary atom site location: structure-invariant direct methods
Least-squares matrix: full	Secondary atom site location: difference Fourier map
*R*[*F*^2^ > 2σ(*F*^2^)] = 0.051	Hydrogen site location: inferred from neighbouring sites
*wR*(*F*^2^) = 0.148	H atoms treated by a mixture of independent and constrained refinement
*S* = 1.03	*w* = 1/[σ^2^(*F*_o_^2^) + (0.070*P*)^2^ + 1.6929*P*] where *P* = (*F*_o_^2^ + 2*F*_c_^2^)/3
7344 reflections	(Δ/σ)_max_ < 0.001
401 parameters	Δρ_max_ = 0.85 e Å^−^^3^
0 restraints	Δρ_min_ = −0.35 e Å^−^^3^

### Fractional atomic coordinates and isotropic or equivalent isotropic displacement parameters (Å^2^)

**Table:**

	*x*	*y*	*z*	*U*_iso_*/*U*_eq_	
N1	0.25623 (8)	0.71175 (10)	0.31330 (8)	0.0251 (2)	
H71	0.2998 (14)	0.6749 (15)	0.3494 (13)	0.034 (5)*	
N2	0.10014 (8)	0.56688 (9)	0.26578 (7)	0.0238 (2)	
C1	0.29863 (11)	0.95171 (13)	0.41663 (10)	0.0338 (3)	
H1A	0.2459	0.9812	0.3774	0.041*	
C2	0.33475 (11)	0.86690 (12)	0.39167 (10)	0.0326 (3)	
H2	0.3869	0.8355	0.4301	0.039*	
C3	0.29836 (12)	0.81760 (12)	0.30658 (10)	0.0341 (3)	
H3A	0.3508	0.8110	0.2777	0.041*	
H3B	0.2495	0.8628	0.2711	0.041*	
C4	0.17100 (10)	0.71680 (12)	0.34936 (10)	0.0287 (3)	
H4A	0.1231	0.7629	0.3138	0.034*	
H4B	0.1889	0.7459	0.4077	0.034*	
C5	0.12940 (10)	0.61023 (12)	0.35209 (9)	0.0279 (3)	
H5A	0.1770	0.5644	0.3883	0.034*	
H5B	0.0741	0.6143	0.3775	0.034*	
C6	0.18554 (10)	0.55568 (12)	0.23334 (9)	0.0267 (3)	
H6A	0.1686	0.5236	0.1761	0.032*	
H6B	0.2310	0.5095	0.2716	0.032*	
C7	0.23147 (11)	0.65942 (12)	0.22794 (9)	0.0285 (3)	
H7A	0.2895	0.6497	0.2072	0.034*	
H7B	0.1877	0.7039	0.1866	0.034*	
C8	0.05330 (9)	0.46543 (11)	0.26749 (9)	0.0234 (3)	
H8	0.0999	0.4178	0.3043	0.028*	
C11	0.33137 (12)	1.00538 (12)	0.49873 (10)	0.0328 (3)	
C12	0.41578 (13)	0.97939 (14)	0.55689 (11)	0.0393 (4)	
H12	0.4549	0.9265	0.5432	0.047*	
C13	0.44279 (15)	1.03042 (18)	0.63443 (12)	0.0502 (5)	
H13	0.4998	1.0115	0.6739	0.060*	
C14	0.38744 (17)	1.10827 (18)	0.65452 (13)	0.0555 (5)	
H14	0.4068	1.1435	0.7074	0.067*	
C15	0.30396 (16)	1.13519 (17)	0.59790 (14)	0.0521 (5)	
H15	0.2657	1.1887	0.6119	0.062*	
C16	0.27599 (13)	1.08397 (14)	0.52044 (12)	0.0407 (4)	
H16	0.2184	1.1027	0.4817	0.049*	
C21	−0.03163 (10)	0.47510 (11)	0.30648 (9)	0.0246 (3)	
C22	−0.04283 (11)	0.40380 (12)	0.36751 (10)	0.0323 (3)	
H22	0.0030	0.3509	0.3849	0.039*	
C23	−0.12068 (13)	0.40914 (14)	0.40346 (11)	0.0397 (4)	
H23	−0.1275	0.3601	0.4454	0.048*	
C24	−0.18792 (12)	0.48540 (14)	0.37840 (12)	0.0388 (4)	
H24	−0.2414	0.4885	0.4024	0.047*	
C25	−0.17702 (11)	0.55719 (13)	0.31830 (11)	0.0352 (3)	
H25	−0.2231	0.6099	0.3011	0.042*	
C26	−0.09910 (10)	0.55295 (12)	0.28272 (10)	0.0294 (3)	
H26	−0.0918	0.6033	0.2420	0.035*	
C31	0.02530 (10)	0.41884 (11)	0.17808 (9)	0.0251 (3)	
C32	0.06680 (12)	0.32722 (12)	0.16047 (11)	0.0337 (3)	
H32	0.1122	0.2937	0.2044	0.040*	
C33	0.04250 (13)	0.28413 (13)	0.07907 (12)	0.0394 (4)	
H33	0.0719	0.2220	0.0676	0.047*	
C34	−0.02374 (13)	0.33119 (14)	0.01544 (11)	0.0386 (4)	
H34	−0.0400	0.3019	−0.0401	0.046*	
C35	−0.06687 (13)	0.42141 (15)	0.03242 (10)	0.0390 (4)	
H35	−0.1138	0.4532	−0.0112	0.047*	
C36	−0.04164 (11)	0.46560 (13)	0.11309 (10)	0.0322 (3)	
H36	−0.0705	0.5284	0.1239	0.039*	
O1	0.63343 (8)	0.37345 (10)	0.53526 (8)	0.0398 (3)	
O2	0.58174 (10)	0.44031 (15)	0.67150 (9)	0.0619 (4)	
O3	0.43582 (10)	0.48104 (13)	0.65686 (9)	0.0557 (4)	
O4	0.19460 (9)	0.32142 (13)	0.44738 (11)	0.0578 (4)	
O5	0.22771 (11)	0.24235 (14)	0.34093 (10)	0.0635 (4)	
O6	0.54743 (11)	0.23538 (12)	0.30218 (9)	0.0525 (4)	
O7	0.66612 (10)	0.28953 (12)	0.40548 (10)	0.0517 (4)	
H7	0.6736	0.3061	0.4569	0.078*	
N3	0.49860 (10)	0.43821 (12)	0.63196 (9)	0.0388 (3)	
N4	0.25126 (10)	0.29137 (13)	0.40744 (10)	0.0436 (4)	
C9	0.57578 (13)	0.27360 (14)	0.37134 (12)	0.0402 (4)	
C41	0.54463 (10)	0.35631 (12)	0.50730 (10)	0.0305 (3)	
C42	0.50991 (11)	0.30575 (12)	0.42633 (10)	0.0321 (3)	
C43	0.41582 (12)	0.28489 (13)	0.39505 (10)	0.0332 (3)	
H43	0.3951	0.2501	0.3421	0.040*	
C44	0.35102 (11)	0.31446 (12)	0.44044 (10)	0.0318 (3)	
C45	0.37853 (11)	0.36483 (12)	0.51759 (10)	0.0310 (3)	
H45	0.3329	0.3856	0.5476	0.037*	
C46	0.47329 (11)	0.38454 (12)	0.55055 (10)	0.0297 (3)	

### Atomic displacement parameters (Å^2^)

**Table:**

	*U*^11^	*U*^22^	*U*^33^	*U*^12^	*U*^13^	*U*^23^
N1	0.0223 (6)	0.0289 (6)	0.0240 (6)	−0.0037 (5)	0.0053 (5)	−0.0023 (5)
N2	0.0211 (5)	0.0270 (6)	0.0237 (5)	−0.0032 (4)	0.0064 (4)	−0.0032 (4)
C1	0.0304 (7)	0.0359 (8)	0.0317 (8)	−0.0039 (6)	0.0011 (6)	−0.0002 (6)
C2	0.0314 (7)	0.0309 (7)	0.0331 (8)	−0.0073 (6)	0.0030 (6)	0.0005 (6)
C3	0.0400 (8)	0.0311 (8)	0.0322 (8)	−0.0112 (6)	0.0105 (6)	−0.0016 (6)
C4	0.0231 (6)	0.0334 (7)	0.0307 (7)	−0.0026 (5)	0.0087 (6)	−0.0078 (6)
C5	0.0251 (7)	0.0345 (7)	0.0254 (7)	−0.0053 (6)	0.0083 (5)	−0.0053 (6)
C6	0.0240 (6)	0.0307 (7)	0.0272 (7)	−0.0035 (5)	0.0095 (5)	−0.0048 (5)
C7	0.0287 (7)	0.0342 (7)	0.0235 (6)	−0.0061 (6)	0.0080 (5)	−0.0042 (6)
C8	0.0205 (6)	0.0252 (6)	0.0246 (6)	0.0003 (5)	0.0058 (5)	0.0008 (5)
C11	0.0354 (8)	0.0313 (7)	0.0310 (7)	−0.0078 (6)	0.0064 (6)	−0.0001 (6)
C12	0.0396 (9)	0.0414 (9)	0.0344 (8)	−0.0059 (7)	0.0040 (7)	0.0005 (7)
C13	0.0517 (11)	0.0614 (12)	0.0325 (9)	−0.0141 (9)	0.0000 (8)	0.0007 (8)
C14	0.0685 (14)	0.0629 (13)	0.0365 (9)	−0.0230 (11)	0.0154 (9)	−0.0156 (9)
C15	0.0600 (12)	0.0464 (11)	0.0552 (12)	−0.0103 (9)	0.0244 (10)	−0.0147 (9)
C16	0.0402 (9)	0.0359 (8)	0.0456 (9)	−0.0052 (7)	0.0095 (7)	−0.0041 (7)
C21	0.0217 (6)	0.0264 (6)	0.0253 (6)	−0.0019 (5)	0.0052 (5)	−0.0023 (5)
C22	0.0320 (7)	0.0331 (8)	0.0340 (8)	0.0033 (6)	0.0120 (6)	0.0052 (6)
C23	0.0436 (9)	0.0399 (9)	0.0423 (9)	0.0008 (7)	0.0236 (8)	0.0059 (7)
C24	0.0347 (8)	0.0390 (9)	0.0497 (10)	−0.0022 (7)	0.0241 (7)	−0.0077 (7)
C25	0.0279 (7)	0.0339 (8)	0.0446 (9)	0.0044 (6)	0.0105 (6)	−0.0057 (7)
C26	0.0281 (7)	0.0280 (7)	0.0325 (7)	0.0014 (6)	0.0084 (6)	0.0003 (6)
C31	0.0231 (6)	0.0258 (6)	0.0279 (7)	−0.0047 (5)	0.0089 (5)	−0.0018 (5)
C32	0.0337 (8)	0.0301 (7)	0.0373 (8)	0.0019 (6)	0.0084 (6)	−0.0025 (6)
C33	0.0452 (9)	0.0304 (8)	0.0451 (9)	−0.0014 (7)	0.0156 (8)	−0.0096 (7)
C34	0.0467 (9)	0.0383 (9)	0.0322 (8)	−0.0107 (7)	0.0122 (7)	−0.0100 (7)
C35	0.0401 (9)	0.0457 (9)	0.0285 (8)	−0.0005 (7)	0.0031 (7)	−0.0022 (7)
C36	0.0321 (7)	0.0341 (8)	0.0300 (7)	0.0026 (6)	0.0066 (6)	−0.0019 (6)
O1	0.0252 (5)	0.0475 (7)	0.0449 (7)	−0.0019 (5)	0.0047 (5)	0.0070 (5)
O2	0.0369 (7)	0.0965 (13)	0.0456 (8)	0.0019 (7)	−0.0034 (6)	−0.0231 (8)
O3	0.0473 (8)	0.0700 (10)	0.0462 (8)	0.0148 (7)	0.0041 (6)	−0.0177 (7)
O4	0.0247 (6)	0.0743 (10)	0.0719 (10)	0.0057 (6)	0.0065 (6)	0.0004 (8)
O5	0.0467 (8)	0.0779 (11)	0.0535 (9)	−0.0096 (8)	−0.0122 (7)	−0.0138 (8)
O6	0.0609 (9)	0.0615 (9)	0.0405 (7)	0.0067 (7)	0.0230 (7)	−0.0021 (6)
O7	0.0400 (7)	0.0633 (9)	0.0579 (8)	0.0010 (6)	0.0236 (6)	−0.0020 (7)
N3	0.0367 (7)	0.0409 (8)	0.0361 (7)	0.0042 (6)	0.0037 (6)	−0.0025 (6)
N4	0.0309 (7)	0.0474 (9)	0.0452 (8)	−0.0006 (6)	−0.0051 (6)	0.0038 (7)
C9	0.0398 (9)	0.0360 (8)	0.0489 (10)	0.0046 (7)	0.0188 (8)	0.0090 (7)
C41	0.0246 (7)	0.0280 (7)	0.0373 (8)	0.0023 (5)	0.0045 (6)	0.0099 (6)
C42	0.0336 (8)	0.0300 (7)	0.0354 (8)	0.0049 (6)	0.0135 (6)	0.0076 (6)
C43	0.0378 (8)	0.0321 (8)	0.0287 (7)	0.0010 (6)	0.0059 (6)	0.0027 (6)
C44	0.0245 (7)	0.0338 (8)	0.0338 (8)	0.0008 (6)	0.0003 (6)	0.0030 (6)
C45	0.0261 (7)	0.0327 (7)	0.0341 (8)	0.0058 (6)	0.0069 (6)	0.0022 (6)
C46	0.0292 (7)	0.0287 (7)	0.0286 (7)	0.0025 (6)	0.0018 (6)	0.0018 (6)

### Geometric parameters (Å, º)

**Table:**

N1—C4	1.4940 (18)	C22—C23	1.395 (2)
N1—C7	1.5009 (18)	C22—H22	0.9500
N1—C3	1.5151 (19)	C23—C24	1.381 (3)
N1—H71	0.89 (2)	C23—H23	0.9500
N2—C6	1.4683 (17)	C24—C25	1.380 (3)
N2—C5	1.4685 (17)	C24—H24	0.9500
N2—C8	1.4826 (17)	C25—C26	1.391 (2)
C1—C2	1.321 (2)	C25—H25	0.9500
C1—C11	1.472 (2)	C26—H26	0.9500
C1—H1A	0.9500	C31—C36	1.388 (2)
C2—C3	1.493 (2)	C31—C32	1.391 (2)
C2—H2	0.9500	C32—C33	1.394 (2)
C3—H3A	0.9900	C32—H32	0.9500
C3—H3B	0.9900	C33—C34	1.372 (3)
C4—C5	1.511 (2)	C33—H33	0.9500
C4—H4A	0.9900	C34—C35	1.384 (3)
C4—H4B	0.9900	C34—H34	0.9500
C5—H5A	0.9900	C35—C36	1.390 (2)
C5—H5B	0.9900	C35—H35	0.9500
C6—C7	1.511 (2)	C36—H36	0.9500
C6—H6A	0.9900	O1—C41	1.2826 (19)
C6—H6B	0.9900	O2—N3	1.226 (2)
C7—H7A	0.9900	O3—N3	1.217 (2)
C7—H7B	0.9900	O4—N4	1.227 (2)
C8—C21	1.5224 (18)	O5—N4	1.224 (2)
C8—C31	1.5279 (19)	O6—C9	1.201 (2)
C8—H8	1.0000	O7—C9	1.315 (2)
C11—C16	1.394 (2)	O7—H7	0.8399
C11—C12	1.397 (2)	N3—C46	1.455 (2)
C12—C13	1.387 (3)	N4—C44	1.453 (2)
C12—H12	0.9500	C9—C42	1.514 (2)
C13—C14	1.377 (3)	C41—C46	1.432 (2)
C13—H13	0.9500	C41—C42	1.442 (2)
C14—C15	1.379 (3)	C42—C43	1.369 (2)
C14—H14	0.9500	C43—C44	1.381 (2)
C15—C16	1.388 (3)	C43—H43	0.9500
C15—H15	0.9500	C44—C45	1.378 (2)
C16—H16	0.9500	C45—C46	1.379 (2)
C21—C22	1.388 (2)	C45—H45	0.9500
C21—C26	1.395 (2)		
			
C4—N1—C7	109.85 (11)	C15—C16—H16	119.6
C4—N1—C3	112.16 (12)	C11—C16—H16	119.6
C7—N1—C3	110.73 (11)	C22—C21—C26	118.69 (13)
C4—N1—H71	107.2 (12)	C22—C21—C8	119.03 (13)
C7—N1—H71	109.6 (12)	C26—C21—C8	122.28 (13)
C3—N1—H71	107.1 (13)	C21—C22—C23	120.60 (15)
C6—N2—C5	107.43 (11)	C21—C22—H22	119.7
C6—N2—C8	110.63 (11)	C23—C22—H22	119.7
C5—N2—C8	110.50 (11)	C24—C23—C22	120.24 (15)
C2—C1—C11	126.91 (15)	C24—C23—H23	119.9
C2—C1—H1A	116.5	C22—C23—H23	119.9
C11—C1—H1A	116.5	C25—C24—C23	119.60 (14)
C1—C2—C3	123.84 (15)	C25—C24—H24	120.2
C1—C2—H2	118.1	C23—C24—H24	120.2
C3—C2—H2	118.1	C24—C25—C26	120.50 (15)
C2—C3—N1	112.36 (12)	C24—C25—H25	119.8
C2—C3—H3A	109.1	C26—C25—H25	119.8
N1—C3—H3A	109.1	C25—C26—C21	120.35 (14)
C2—C3—H3B	109.1	C25—C26—H26	119.8
N1—C3—H3B	109.1	C21—C26—H26	119.8
H3A—C3—H3B	107.9	C36—C31—C32	118.51 (14)
N1—C4—C5	110.38 (12)	C36—C31—C8	121.53 (13)
N1—C4—H4A	109.6	C32—C31—C8	119.96 (13)
C5—C4—H4A	109.6	C31—C32—C33	120.64 (15)
N1—C4—H4B	109.6	C31—C32—H32	119.7
C5—C4—H4B	109.6	C33—C32—H32	119.7
H4A—C4—H4B	108.1	C34—C33—C32	120.22 (16)
N2—C5—C4	110.34 (12)	C34—C33—H33	119.9
N2—C5—H5A	109.6	C32—C33—H33	119.9
C4—C5—H5A	109.6	C33—C34—C35	119.77 (15)
N2—C5—H5B	109.6	C33—C34—H34	120.1
C4—C5—H5B	109.6	C35—C34—H34	120.1
H5A—C5—H5B	108.1	C34—C35—C36	120.18 (16)
N2—C6—C7	110.95 (12)	C34—C35—H35	119.9
N2—C6—H6A	109.4	C36—C35—H35	119.9
C7—C6—H6A	109.4	C31—C36—C35	120.65 (15)
N2—C6—H6B	109.4	C31—C36—H36	119.7
C7—C6—H6B	109.4	C35—C36—H36	119.7
H6A—C6—H6B	108.0	C9—O7—H7	109.5
N1—C7—C6	111.07 (11)	O3—N3—O2	122.94 (16)
N1—C7—H7A	109.4	O3—N3—C46	117.98 (14)
C6—C7—H7A	109.4	O2—N3—C46	119.07 (14)
N1—C7—H7B	109.4	O5—N4—O4	122.97 (16)
C6—C7—H7B	109.4	O5—N4—C44	118.25 (16)
H7A—C7—H7B	108.0	O4—N4—C44	118.78 (15)
N2—C8—C21	110.99 (11)	O6—C9—O7	122.73 (17)
N2—C8—C31	110.55 (11)	O6—C9—C42	122.32 (17)
C21—C8—C31	111.42 (11)	O7—C9—C42	114.95 (16)
N2—C8—H8	107.9	O1—C41—C46	124.92 (15)
C21—C8—H8	107.9	O1—C41—C42	120.16 (15)
C31—C8—H8	107.9	C46—C41—C42	114.92 (13)
C16—C11—C12	118.36 (16)	C43—C42—C41	121.77 (14)
C16—C11—C1	119.29 (15)	C43—C42—C9	116.64 (15)
C12—C11—C1	122.35 (16)	C41—C42—C9	121.59 (15)
C13—C12—C11	120.39 (18)	C42—C43—C44	120.03 (15)
C13—C12—H12	119.8	C42—C43—H43	120.0
C11—C12—H12	119.8	C44—C43—H43	120.0
C14—C13—C12	120.41 (19)	C45—C44—C43	121.62 (14)
C14—C13—H13	119.8	C45—C44—N4	118.47 (15)
C12—C13—H13	119.8	C43—C44—N4	119.91 (15)
C13—C14—C15	120.06 (18)	C44—C45—C46	118.90 (14)
C13—C14—H14	120.0	C44—C45—H45	120.5
C15—C14—H14	120.0	C46—C45—H45	120.5
C14—C15—C16	119.9 (2)	C45—C46—C41	122.74 (14)
C14—C15—H15	120.0	C45—C46—N3	116.77 (14)
C16—C15—H15	120.0	C41—C46—N3	120.48 (14)
C15—C16—C11	120.85 (18)		
			
C11—C1—C2—C3	−179.12 (15)	N2—C8—C31—C36	−64.73 (17)
C1—C2—C3—N1	−114.54 (17)	C21—C8—C31—C36	59.19 (17)
C4—N1—C3—C2	63.88 (17)	N2—C8—C31—C32	115.63 (14)
C7—N1—C3—C2	−173.02 (13)	C21—C8—C31—C32	−120.45 (14)
C7—N1—C4—C5	54.46 (16)	C36—C31—C32—C33	0.8 (2)
C3—N1—C4—C5	178.06 (12)	C8—C31—C32—C33	−179.55 (14)
C6—N2—C5—C4	63.12 (15)	C31—C32—C33—C34	−0.8 (3)
C8—N2—C5—C4	−176.12 (11)	C32—C33—C34—C35	−0.4 (3)
N1—C4—C5—N2	−60.74 (15)	C33—C34—C35—C36	1.5 (3)
C5—N2—C6—C7	−61.67 (15)	C32—C31—C36—C35	0.3 (2)
C8—N2—C6—C7	177.64 (11)	C8—C31—C36—C35	−179.31 (14)
C4—N1—C7—C6	−53.14 (16)	C34—C35—C36—C31	−1.5 (3)
C3—N1—C7—C6	−177.57 (12)	O1—C41—C42—C43	179.06 (15)
N2—C6—C7—N1	57.82 (16)	C46—C41—C42—C43	−1.4 (2)
C6—N2—C8—C21	176.26 (11)	O1—C41—C42—C9	−1.3 (2)
C5—N2—C8—C21	57.42 (14)	C46—C41—C42—C9	178.26 (14)
C6—N2—C8—C31	−59.58 (14)	O6—C9—C42—C43	2.9 (2)
C5—N2—C8—C31	−178.42 (11)	O7—C9—C42—C43	−176.34 (15)
C2—C1—C11—C16	−169.51 (17)	O6—C9—C42—C41	−176.79 (16)
C2—C1—C11—C12	9.5 (3)	O7—C9—C42—C41	4.0 (2)
C16—C11—C12—C13	0.5 (3)	C41—C42—C43—C44	1.4 (2)
C1—C11—C12—C13	−178.46 (16)	C9—C42—C43—C44	−178.21 (14)
C11—C12—C13—C14	−0.9 (3)	C42—C43—C44—C45	−0.2 (2)
C12—C13—C14—C15	0.8 (3)	C42—C43—C44—N4	−179.44 (15)
C13—C14—C15—C16	−0.3 (3)	O5—N4—C44—C45	−176.60 (17)
C14—C15—C16—C11	−0.1 (3)	O4—N4—C44—C45	3.2 (2)
C12—C11—C16—C15	0.0 (3)	O5—N4—C44—C43	2.7 (2)
C1—C11—C16—C15	179.02 (17)	O4—N4—C44—C43	−177.57 (16)
N2—C8—C21—C22	−133.38 (14)	C43—C44—C45—C46	−1.0 (2)
C31—C8—C21—C22	102.95 (15)	N4—C44—C45—C46	178.23 (14)
N2—C8—C21—C26	46.96 (17)	C44—C45—C46—C41	1.0 (2)
C31—C8—C21—C26	−76.71 (17)	C44—C45—C46—N3	179.78 (14)
C26—C21—C22—C23	0.8 (2)	O1—C41—C46—C45	179.67 (15)
C8—C21—C22—C23	−178.82 (15)	C42—C41—C46—C45	0.1 (2)
C21—C22—C23—C24	0.3 (3)	O1—C41—C46—N3	1.0 (2)
C22—C23—C24—C25	−0.8 (3)	C42—C41—C46—N3	−178.57 (13)
C23—C24—C25—C26	0.2 (3)	O3—N3—C46—C45	−15.1 (2)
C24—C25—C26—C21	0.9 (2)	O2—N3—C46—C45	166.34 (17)
C22—C21—C26—C25	−1.4 (2)	O3—N3—C46—C41	163.64 (16)
C8—C21—C26—C25	178.21 (14)	O2—N3—C46—C41	−14.9 (2)

### Hydrogen-bond geometry (Å, º)

**Table:**

*D*—H···*A*	*D*—H	H···*A*	*D*···*A*	*D*—H···*A*
N1—H71···O1^i^	0.89 (2)	1.99 (2)	2.8105 (18)	153.5 (17)
N1—H71···O2^i^	0.89 (2)	2.36 (2)	3.037 (2)	132.4 (16)
O7—H7···O1	0.84	1.75	2.507 (2)	149
C3—H3*A*···O6^ii^	0.99	2.40	3.341 (2)	160
C32—H32···O5	0.95	2.52	3.453 (2)	166

Symmetry codes: (i) −*x*+1, −*y*+1, −*z*+1; (ii) −*x*+1, *y*+1/2, −*z*+1/2.
